# *Aspalathus linearis* (Rooibos) and Agmatine May Act Synergistically to Beneficially Modulate Intestinal Tight Junction Integrity and Inflammatory Profile

**DOI:** 10.3390/ph15091097

**Published:** 2022-09-01

**Authors:** L. Pretorius, C. Smith

**Affiliations:** 1 Department of Physiological Sciences, Faculty of Science, Stellenbosch University, Stellenbosch 7600, South Africa; 2 Division of Clinical Pharmacology, Department of Medicine, Faculty of Medicine and Health Sciences, Stellenbosch University, Cape Town 7500, South Africa

**Keywords:** trace amines, occludin, ρ-tyramine, 17β-estradiol, prostaglandin E2, HT-29s

## Abstract

In order to promote gastrointestinal health, significant increases in the prevalence of gastrointestinal disorders should be paralleled by similar surges in therapeutics research. Nutraceutical interventions may play a significant role in patient management. The current study aimed to determine the potential of *Aspalathus linearis* (rooibos) to prevent gastrointestinal dysregulation resulting from high-dose trace-amine (TA) exposure. Considering the substantial female bias in functional gastrointestinal disorders, and the suggested phytoestrogenicity of rooibos, the study design allowed for a comparison between the effects of an ethanol extract of green rooibos and 17β-estradiol (E2). High levels of ρ-tyramine (TYR) and agmatine (AGM), but not β-phenethylamine (PEA) or tryptamine (TRP), resulted in prostaglandin E2 (PGE2) hypersecretion, increased tight-junction protein (TJP; occludin and ZO-1) secretion and (dissimilarly) disrupted the TJP cellular distribution profile. Modulating benefits of rooibos and E2 were TA-specific. Rooibos pre-treatment generally reduced IL-8 secretion across all TA conditions and prevented PGE2 hypersecretion after exposure to both TYR and AGM, but was only able to normalise TJP levels and the distribution profile in AGM-exposed cells. In contrast, E2 pre-treatment prevented only TYR-associated PGE2 hypersecretion and TJP dysregulation. Together, the data suggest that the antioxidant and anti-inflammatory effects of rooibos, rather than phytoestrogenicity, affect benefits illustrated for rooibos.

## 1. Introduction

Functional gastrointestinal disorders (FGIDs) are highly prevalent (approximately 40% of the global population) conditions that considerably reduce quality of life, placing enormous economic burdens on healthcare systems worldwide [1,2,3]. Of importance, many FGIDs have a reported female predominance [4,5,6,7], suggesting a potential estrogen-driven vulnerability to gastrointestinal disturbances. Current therapeutic strategies (including drug and biological treatments) are not yet addressing this aspect, and have been associated with adverse systemic effects such as adverse cardiovascular events [8,9], which may exacerbate patient discomfort. As such, the use of functional foods/nutraceuticals is gaining research interest in the promotion of gastrointestinal health while minimising adverse effects [10,11,12,13,14,15,16].

Of particular interest to the current study context, rooibos herbal tea (brewed from unfermented or fermented *Aspalathus linearis*), is a widely consumed traditional South African tisane that has already been suggested as a functional food [17,18] due to its substantial and unique blend of bioactive polyphenols which have been linked to potent antioxidant actions [19,20,21]. Preparation of the rooibos extracts—e.g., choice of solvent and processing (fermentation or not)—has been demonstrated to affect potency of the antioxidant effect achieved, with green (unfermented) extracts showing greater antioxidant effect. Mechanisms reported mainly include hydrogen-ion donation and superoxide quenching [22]. In terms of the specific ethanolic green rooibos extract (GRE) employed in the current study, similar mechanisms have been reported. For example, we demonstrated in the context of neuroprotection, that GRE—which has an ORAC value of 8.1 µmol TE/mg [23]—was able to quench both synthetic (DPPH) and physiological (superoxide) radicals to protect neuronal cells from LPS-induced oxidative damage [23].

In terms of promoting gut health specifically, scant literature suggests beneficial actions (anti-inflammatory, anti-nociceptive, anti-spasmodic) of several bioactive constituents of rooibos, such as iso-orientin [24,25], quercetin [26,27,28,29,30], chrysoeriol, orientin, vitexin and rutin [31] in various in vitro and in vivo models, which could potentially reduce FGIDs symptomology. We have recently expanded on this body of literature by demonstrating that rooibos—in a manner very dependent on extraction/processing—beneficially modulated the secretory profile of gut microbes [32]. In the same study, 17β-estradiol (E2)-associated changes in microbial secretory function were largely negated by rooibos, suggesting that rooibos supplementation may stabilize gut signalling profile in females, which may aid patient management. Related to this, a moderate phytoestrogenicity has been reported for rooibos [33,34,35], which makes it an interesting candidate nutraceutical for investigation in female-predominant disorders, such as irritable bowel syndrome.

According to Wan et al. (2019), two crucial factors determine gut health, namely the gut microbiota and the intestinal epithelial barrier. Indeed, in line with our current hypothesis of trace-aminergic involvement in exaggeration of gastrointestinal symptomology [36], we have recently demonstrated the deleterious effects of high trace-amine (TA) load on intestinal epithelial tight-junction proteins (TJPs) and inflammatory status [37]. In this regard, we propose that the known poor absorption and low systemic bioavailability of rooibos and some of its polyphenolic constituents [38,39,40] may not be a significant limitation if beneficial modulation of rooibos occurs directly at an intestinal epithelial level. In support of this notion, a recent review suggested that dietary polyphenols can be metabolised by gut microbes, which could exert beneficial effects toward the gut epithelium, as well as systemically [41]. In terms of intestinal-barrier integrity and intestinal inflammation, protective effects of flavonoids have been described in several studies utilising both in vitro and in vivo models [13,42,43,44], suggesting nutraceutical interventions may ameliorate gastrointestinal dysfunction and inflammation, which underpins many gastrointestinal conditions. However, to the best of our knowledge, no studies have investigated the effects of rooibos on intestinal cells.

As such, to determine the potential of rooibos to promote gut health, this study aimed to investigate the use of a selected (refer to supplemental material) rooibos preparation (ethanol extract of green rooibos; GRE) as pre-treatment to protect against TA-induced TJP disruption, with concurrent inflammatory status assessments in colon adenocarcinoma (HT-29) cells. In particular, the specific TAs β-phenethylamine (PEA), tryptamine (TRP), ρ-tyramine (TYR) and agmatine (AGM) were utilised, given their known roles in gastrointestinal physiology [45,46,47,48,49] and/or gastrointestinal disorders [50,51,52]. In addition, inflammatory assessments included a panel of cytokines and prostaglandin E2 (PGE2) to extrapolate current findings to the known inflammatory profile associated with FGIDs [53,54]. To enable probing potential phytoestrogenicity of rooibos, a parallel experiment using E2 pre-treatment instead of rooibos was also included.

## 2. Results

### 2.1. Rooibos and Estradiol Have Differential Protective Effects on Tight-Junction Integrity following Trace-Amine Exposure

**Figure 1:**
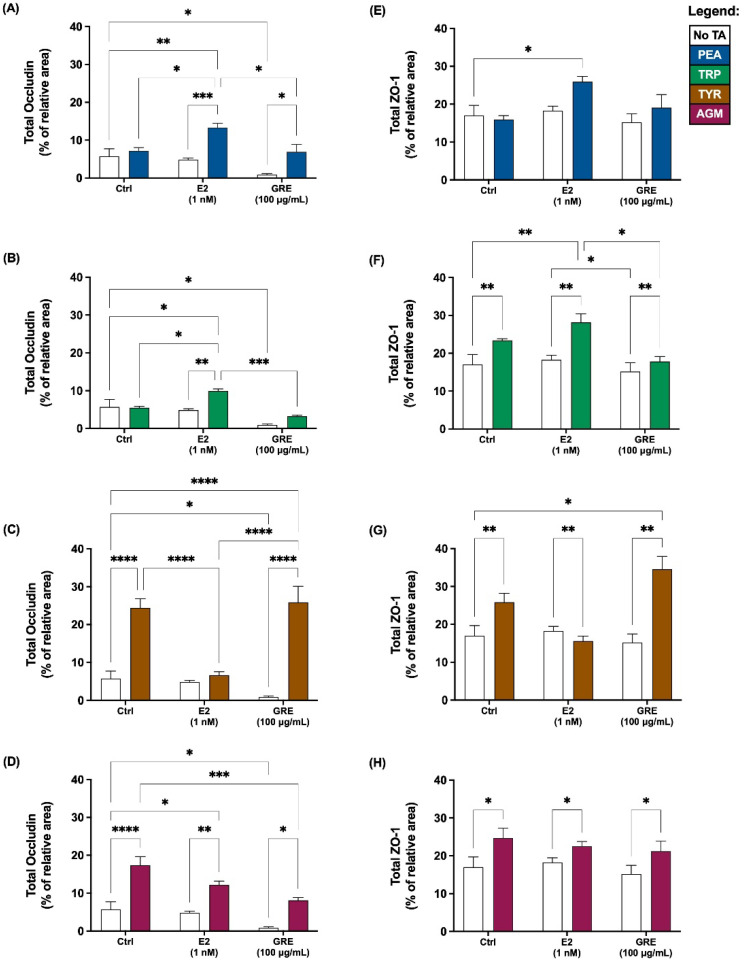
Effects of green rooibos extract (GRE) and 17β-estradiol (E2) on the percentage of relative area of imaged HT-29 cell monolayer that stained positive for tight-junction proteins occludin (**A**–**D**) and ZO-1 (**E**–**H**) following selected TA exposure (200 ng/mL): (**A**,**E**) β-phenethylamine (PEA), (**B**,**F**) tryptamine (TRP), (**C**,**G**) ρ-tyramine (TYR) and (**D**,**H**) agmatine (AGM). Data are represented as mean (% of total imaged area) ± SEM. Statistical analysis: 2-way ANOVAs with Tukey’s multiple comparison tests: * = *p* < 0.05, ** = *p* < 0.01, *** = *p* < 0.001, **** = *p* < 0.0001.

**Figure 2:**
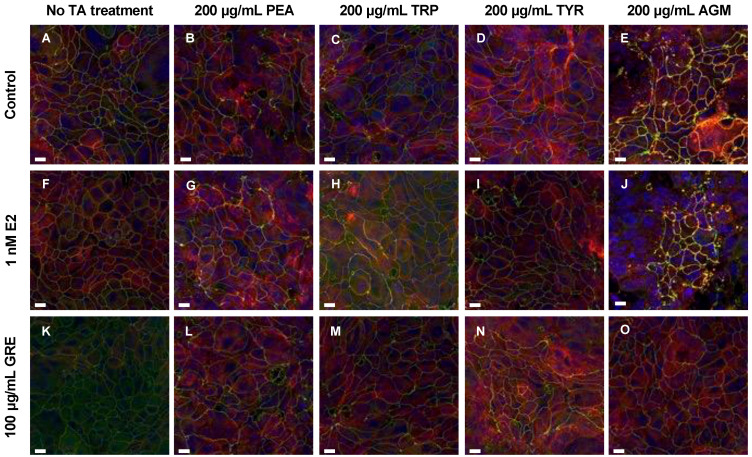
Representative fluorescent micrographs showing the effects of trace amines (TA, frames (**B**–**E**)), green rooibos extract (GRE, frame (**F**)), 17β-estradiol (E2, frame (**K**)) and combined treatments (frames (**G**–**J**,**L**–**O**)) vs. control (**A**) on tight-junction proteins in HT-29 cell monolayers. Green signal = ZO-1, red signal = occludin, yellow signal = colocalized ZO-1 and occludin signal, and blue signal = Hoechst. All fluorescent images are maximum intensity projections of acquired z-stacks, imaged using the 60× oil immersion objective. Scale bar = 10 μm. Abbreviations: PEA: β-phenethylamine, TRP: tryptamine, TYR: ρ-tyramine, AGM: agmatine.

TJP (occludin and zona occludens-1 (ZO-1)) levels and distribution profiles were assessed in HT-29 colon adenocarcinoma monolayers as an indication of gut barrier epithelial integrity. These data are represented both quantitatively (Figure 1) and qualitatively (Figure 2). In terms of conditioning of cells with either E2 or GRE, in the absence of TA-exposure, both treatments maintained—if not enhanced—normal cellular morphology, although no changes in total ZO-1 expression were evident (Figure 1). More specifically, E2 seemed to have a beneficial modulatory effect on cell size and shape, resulting in a more uniformly sized cell monolayer (Figure 2F), while GRE treatment reduced occludin signal significantly (Figure 2K) compared to control conditions (*p* < 0.05).

In the presence of TAs and without pre-treatment (control), TJPs were differentially disrupted (clustered or dispersed and irregularly localised) and cellular morphology was altered to varying degrees by all TAs included. Exposure to PEA and TRP seemed the least detrimental, while AGM exposure induced the most significant damage to monolayer integrity (Figure 2E). Although PEA treatment did not alter TJP levels significantly (Figure 1A,E), qualitatively a mild intracellular redistribution of occludin, as well as larger areas without ZO-1 expression, was observed (Figure 2B). TRP treatment only altered cellular morphology slightly (Figure 2C), despite significantly increased total ZO-1 expression levels (Figure 1F; *p* < 0.01 vs. control). In contrast, of all TAs assessed, TYR treatment significantly increased occludin (Figure 1C; *p* < 0.01) the most. This ≈ 20% increase was associated with significant internalisation and clustering of occludin (Figure 2D). TYR treatment also significantly increased ZO-1 expression (Figure 1G; *p* < 0.0001), which appeared less organised than the control. AGM treatment prominently increased total occludin (Figure 1D; *p* < 0.05) and ZO-1 expression levels (Figure 1H; *p* < 0.05). Notably, AGM treatment clustered TJPs intensely at membrane junctions in areas of intact monolayer—evidenced by the intense yellow signal in some, but not all, cells (Figure 2E)—but also resulted in interspersed areas where TJP disruption was evident. These different outcomes for TYR and AGM in terms of TJP profile, highlight the importance of assessing both expression levels and cellular distribution profiles of TJPs.

Moreover, the effects of E2 pre-treatment on the cellular response to TA-exposure was variable. In the context of PEA and TRP treatment, the presence of E2 significantly increased occludin (Figure 1A,B) and, to a lesser extent, ZO-1 expression (Figure 1E,F). These results are reflected in the respective representative images which depict increased occludin clustering and internalization (Figure 2G) and higher levels of ZO-1 signal (Figure 2H). Markedly, following TYR treatment, E2 pre-treated monolayers had normalised occludin and ZO-1 levels (Figure 1C,G). Indeed, the E2 and TYR group had significantly lower occludin than both other TYR groups (*p* < 0.0001 for both), which translated to regular TJP distribution and localization (Figure 2I), resembling a control profile. In terms of AGM, no obvious E2 effect was apparent.

Turning attention to rooibos, despite the significant dampening effect of GRE pre-treatment on occludin levels in the absence of TAs, GRE pre-treatment was unable to prevent the occludin response to TA exposure, with the exception of the response to AGM exposure, which was significantly inhibited (Figure 1D). In this context, cellular morphology was generally maintained, and TJP distribution was visually similar to control cells in the absence of TA treatment (Figure 2O). Regarding PEA, TRP and TYR treatment, GRE pre-treatment had no effect on absolute TJP levels, however, cellular morphology in the combination TYR group appeared somewhat modulated (Figure 2N). Taken together, both E2 and GRE pre-treatment had very specific effects, which suggest complex, yet different mechanisms of actions.

### 2.2. Rooibos and Estradiol Differentially Modulate Prostaglandin E2 Secretion following Trace-Amine Exposure

**Figure 3:**
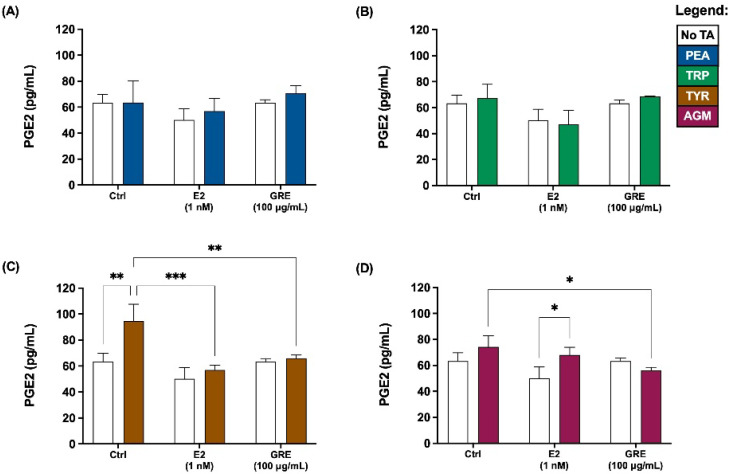
Effects of green rooibos extract (GRE) and 17β-estradiol (E2) on the concentration of prostaglandin E2 (PGE2) secreted by HT-29 cell monolayers following selected TA-exposure (200 ng/mL): (**A**) β-phenethylamine (PEA), (**B**) tryptamine (TRP), (**C**) ρ-tyramine (TYR) and (**D**) agmatine (AGM). Data are represented at mean ± SD, n = 3. Statistical analysis: 2-way ANOVAs with Tukey’s multiple comparison tests: * = *p* < 0.05, ** = *p* < 0.01, *** = *p* < 0.001.

In terms of assessment of inflammatory profile, prostaglandin E2 (PGE2) and inflammatory cytokine levels were determined. In the absence of TA-exposure, neither E2 nor GRE pre-treatment affected PGE2 secretion by HT-29 cells (Figure 3). Similar to the TJP data, PEA and TRP exposure did not result in increased PGE2 secretion, with no apparent effect of either E2 or GRE pre-treatment in this context (Figure 3A,B). In contrast, TYR-exposure under control conditions, increased PGE2 levels most significantly (*p* < 0.01 vs. control) of all TA assessed (Figure 3C). Both E2 and GRE prevented this increase, maintaining PGE2 at baseline levels. However, while this modulatory effect on TYR-induced PGE2 secretion was mediated by both E2 and GRE, from representative micrographs of these cell monolayers, only the effects from E2 seemed to translate into a more beneficial outcome in terms of maintenance of TJP integrity and cellular morphology. Lastly, in response to AGM exposure, a significant but mild PGE2 response was only evident in the presence of E2, while GRE significantly reduced PGE2 response vs. the no pre-treatment AGM-exposed condition (Figure 3D; *p* < 0.05).

### 2.3. Rooibos, but Not Estradiol, Differentially Modulates IL-8 Secretion following Trace-Amine Exposure

**Figure 4:**
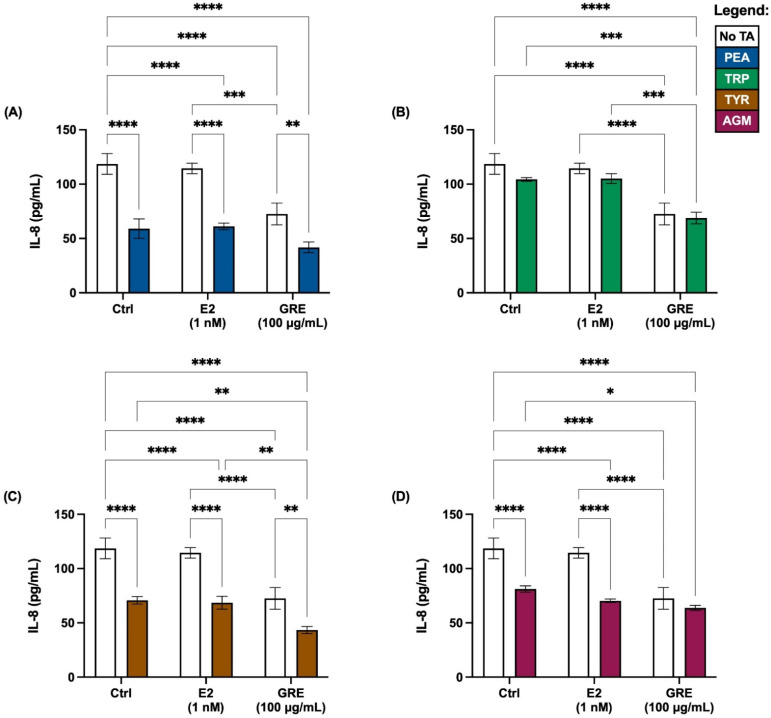
Effects of green rooibos extract (GRE) and estradiol (E2) on the concentration of interleukin-8 (IL-8) secreted by HT-29 cell monolayers following selected TA-exposure (200 ng/mL): (**A**) β-phenethylamine (PEA), (**B**) tryptamine (TRP), (**C**) ρ-tyramine (TYR) and (**D**) agmatine (AGM). The data are represented at mean ± SD, n = 3. Statistical analysis: 2-way ANOVAs with Tukey’s multiple comparison tests: * = *p* < 0.05, ** = *p* < 0.01, *** = *p* < 0.001, **** = *p* < 0.0001.

In terms of cytokine secretion, the overall cellular cytokine response was quite low, with only IL-8 secreted at detectable levels (Figure 4). All TAs employed seemed to reduce the IL-8 response, although not reaching statistical significance for TRP. In the absence of TA treatment, GRE pre-treatment reduced the IL-8, while in the presence of TA its effect was maintained. In contrast, E2 pre-treatment did not seem to have an effect on IL-8 release, suggesting that E2 and GRE are independent role players in this context.

## 3. Discussion

Gastrointestinal epithelia are exposed to numerous exogenous (dietary, microbial and medicinal) metabolites/compounds and endogenous stimuli (inflammatory, hormonal and neural), which can influence gastrointestinal homeostasis. For the epithelial cells to both absorb necessary nutrients and prevent the entry of potentially harmful microbes or dietary antigens, dynamic regulation of intestinal-barrier permeability is crucial. In this context, polyphenol-rich nutraceuticals have seen a dramatic increase in their use as dietary supplements. This is likely due to their widely reported protection against oxidative damage and inflammation, and beneficial modulation of intestinal-barrier integrity [13,42,43,44,55,56], which supports a role for them in management of chronic inflammatory conditions associated with increased intestinal permeability.

Current data yielded the following main novel findings: (i) pre-treatment of HT-29 cells with E2 or GRE both improved cellular ultrastructure, albeit differently; (ii) exposure to TAs generally suppressed the inflammatory response in terms of inflammatory cytokine (IL-8) secretion, although TYR (and to a lesser degree AGM) elicited a significant PGE2 response, which was associated with increased total levels of and altered TJP distribution; (iii) E2 pre-treatment prevented the PGE2 response to TYR only, normalising TJP levels and distribution; while (iv) GRE prevented TA-induced PGE2 and IL-8 secretion in both TYR and AGM, but was only able to normalise TJP levels and the distribution profile for AGM.

Current data illustrate that pre-treatment with both E2 and GRE improved HT-29 monolayer morphology, which adds to literature reporting known beneficial modulation of tight-junction barriers in this context. In the context of E2, this outcome was potentially mediated by estrogen receptor (ER)-β and/or G protein-coupled ER 1(GPER) interactions—both of which have been reported in HT-29 cells [57,58,59]. This interpretation is in line with literature describing destabilization of cellular integrity and cytoarchitecture as a feature of ERβ-/- mice [59], suggesting that activation of ERβ by E2 promoted uniform cellular architecture. This notion also aligns with exaggerated gastrointestinal symptomology during menses, when E2 levels are at their lowest [60]. In the context of rooibos, however, literature regarding its effects on TJPs is sparse and usually in the context of blood–brain-barrier (BBB) assessments. To our knowledge, the results demonstrated in this study are the first to inform on the potential of rooibos to beneficially modulate colonic TJPs following TA (AGM specifically) exposure. According to literature, long-term consumption of fermented rooibos tea was found to protect against BBB disruption and brain oedema following ischemic injury in rats [61]. Similarly, Fisher et al. (2020) reported protective effects of an aspalathin-rich rooibos tincture in an in vitro BBB model. Notably this study utilised bEnd5 cells, which do not express the TJP occludin [62]. In our study, an ethanol extract of green rooibos decreased the total occludin signal significantly under baseline conditions (absence of TA treatment), which, in the context of the report from Fisher et al. (2020), suggests more than one potential mechanism of action for rooibos-related beneficial modulation of tight-junction barriers. Importantly, this downregulation of total absolute occludin expression levels did not compromise cellular integrity or morphology. A potential explanation for this finding is related to rooibos uptake and transport in the gut. In this regard, it has been strongly suggested that aspalathin—the major constituent of GRE used—is potentially transported paracellularly [63], motivating that the decrease in occludin observed may be due to an enhancement of paracellular transport in this context, although this requires more thorough investigations to elucidate the actual mechanism at play.

In terms of TA-induced effects on inflammatory status; although the levels of PGE2—as a prominent prostanoid—seemed to associate with changes in TJP status induced by TA-exposure (specifically TYR and AGM) in this study; cytokine analyses revealed a relatively supressed inflammatory response following TA treatment across the board. However, this seeming contradiction to the increased PGE2 levels is in line with literature. For example, according to Kelly et al. (2015), a feed-forward cycle exists between gut-barrier dysfunction and inflammatory processes. In this context, the authors suggested that an increase in gut permeability, which is generally associated with TJP disruption, could precede mucosal inflammation to stimulate an inflammatory response [64]. In addition, in a similar model, increases in PGE2 was reported to generally precede increases in IL-8 levels [65]. Once produced from membrane phospholipids, PGE2 is secreted, either via passive diffusion or active transport, to exert para- or autocrine effects—which may explain the higher PGE2 levels observed.

Current data implicate TYR especially in PGE2 stimulation. This has specific relevance to gastrointestinal disorders, as elevated levels of faecal TYR were identified as a differential biomarker in patients with inflammatory bowel disease in a cross-sectional study [52] and TYR has demonstrated cellular cytotoxicity at high doses [37,66]. Furthermore, we and others have demonstrated that TYR is an abundant microbial metabolite [32,67,68]. Of particular relevance, TYR formation via L-tyrosine decarboxylation is a well-known characteristic of lactic acid bacteria (LAB) [69], which are commonly consumed as probiotic supplements. Considering TYR as a microbial metabolite, as well as the report by Luqman et al. (2018) that demonstrated promotion of bacterial adherence and subsequent internalization (HT-29s) in the presence of TYR, a low or delayed cytokine response may be in line with microbial adaptations to evade host immune systems [70]. Furthermore, in terms of additional mechanisms that can potentially explain the low cytokine response, despite the generally robust PGE2 response, we hypothesise that occludin-related congestion of the golgi complex [71]—via caveolin-1-dependent mechanisms—may have caused delayed cytokine secretion and may potentially explain the discrepancy between the PGE2 and IL-8 results. In support of this interpretation, our representative micrographs depicted prominent occludin redistribution following TA treatment in general, with most extensive internalization in the presence of TYR. Importantly, caveolin proteins associate with the golgi complex to facilitate transport from the plasma membrane into the cell [72,73]. As such, endocytosis of occludin in this context is likely mediated by caveolin-1 [74], which forms caveolae (plasma membrane invaginations), and is reportedly essential for immune-mediated TJP regulation. Furthermore, in general, caveolin-1 has been reported to limit the inflammatory response [72]. In support of this, LPS and other microbial products have been shown to activate caveolin, which may assist their (microbial) entry into cells/across barriers when there is compromised TJP integrity [72,75]. Certainly, some pathogens exploit caveolae as a route of internalisation that would allow their survival, since it avoids the lysosomal pathway [76]. In fact, caveolin-1 expression increased the susceptibility of M-cells to Salmonella infection [77], implicating caveolin-1 in the gateway of microbial pathogen internalization. While this mechanism of action was not confirmed in the current study, it may—at least in part—explain the inflammatory profile reported here. As such, it would be interesting, in future studies, to assess potential links between caveolin-1, occludin internalisation and TYR.

Despite the relatively similar effects of TYR and AGM—in line with our previous work [37]—in altering both PGE and IL-8 levels and increasing total TJP concentrations, an important differentiating factor in these effects is related, in particular, to the distribution of occludin. In the case of TYR, as mentioned previously, extensive occludin internalisation (i.e., loss of colocalisation with ZO-1) was observed, while exposure to AGM promoted ZO-1 and occludin colocalisation in areas of intact monolayer, but with apparent loss of TJP expression in other areas. In the current study, the inclusion of pre-treatment conditions (E2 and GRE) serves to illustrate potential differences in mechanisms of actions between TYR and AGM. For example, in the context of E2 pre-treatment, E2 prevented TYR-induced PGE2 increases, normalising TJP levels and cellular distribution, particularly that of occludin. While the precise mechanism of action by which TYR disrupts TJPs is unknown, we suggest—at least in part—that hydrogen peroxide, as a by-product of oxidation of TYR via monoamine oxidase (MAO), may induce oxidative stress and damage that could disrupt junctional proteins and integrity. Indeed, the intestine is predominated (<80%) by MAO-A isoforms [78], by which TYR and other TAs (but not PEA, which is highly selective for MOA-B) are deaminated. In addition, a study investigating the effect of MAO substrates on endogenous prostaglandin synthesis in rat-brain homogenates reported that the presence of tyrosine (the precursor L-amino acid to TYR) caused a 2-fold increase in cyclooxygenase activity, increasing PGE2 levels significantly [79], suggesting that the hydrogen peroxide formed during amine degradation stimulated prostaglandin synthesis. While it should be considered that TYR-related internalization of occludin may be mediated by more complex mechanisms, it has been reported that PGE2 may disrupt intestinal epithelial-barrier function, and specifically redistribute occludin towards intracellular locations in vitro [80]. Regarding the preventative E2 effect in this context, the anti-inflammatory effects of estrogen or ERβ agonists have been well described in models of chronic intestinal inflammation [58,81,82,83]. Specifically, E2 is reported to mitigate oxidative damage caused by hydrogen peroxide in several tissue types [84,85,86,87], including in HT-29s, where E2 treatment prevented oxidative damage of the mucus layer, and reduced apoptosis and permeability following hydrogen peroxide challenge [88]. Taken together, if E2 pre-treatment quenched TYR-related hydrogen peroxide, the subsequent increase in PGE2 and occludin redistribution could have been alleviated, as seen in current data. The fact that the (known antioxidant) rooibos pre-treatment was also able to reduce the PGE2 response—although not the normalisation of TJP profile in presence or TYR, supports a role for antioxidants in this context, but also suggests that more than one mechanisms is at play. The relative importance of a direct antioxidant response of E2 (as described above) vs. other mechanisms—such as potential ERβ and/or GPER interactions—in the context of TYR-associated risk remains to be elucidated. Regardless, in terms of the practical implications of current data, the fact that the presence of E2 seems to negate TYR-induced epithelial disruptions, suggests that female patients with elevated gut TYR levels could potentially have significantly exaggerated gastrointestinal symptomology during menses. This fact, as well as the high proportion of TYR secreted by LAB, as already mentioned, also cautions against prescription of these bacteria as probiotic supplements in FGID.

Moving on to address the different outcome to AGM exposure, the effects of rooibos suggest that redox balance is hugely important in optimising effects of AGM, which in the literature is mostly ascribed beneficial roles, albeit at much lower levels. Taking a closer look at the effects of AGM, the literature generally suggests a neuroprotective effect [89], particularly in the context of BBB stabilization and AGM-related reductions in BBB permeability [90]. Importantly, in the current study, representative micrographs reflected increases in ZO-1 and occludin colocalization along the cell membrane, which we suggest may depict an attempt to promote TJP integrity, which would be in line with the mentioned beneficial effects of AGM on the BBB.

Despite this favourable assessment of AGM function on TJPs, the interspersed areas lacking TJP suggest breakdown of cellular membrane integrity—a less desired outcome. However, given the fact that rooibos normalised this profile, we would like to argue that, for two reasons, this negative outcome is an artefact of the cellular model used. Firstly, it is unlikely that AGM was metabolised into its downstream polyamine metabolites (putrescine, spermidine and spermine), as would normally happen in vivo. Indeed, exogenous AGM accumulation inside HT-29s, with minimal subsequent catabolism—ascribed to AGM-associated downregulation of ornithine decarboxylase activity and expression—has been reported [91]. Thus, at high doses, AGM may have directly resulted in epithelial apoptosis. This interpretation is in line with reports of AGM enhancing the release of pro-apoptotic pro-oxidant factors, such as cytochrome c, to potentially induce apoptosis via selective permeabilization of the outer mitochondrial membrane [92].

Secondly, the relative absence of AGM-associated polyamines in the cellular model has further significance. These polyamines—which are non-enzymatic antioxidants [93,94]—have been reported to improve gastrointestinal epithelial integrity and restoration by enhancing TJP expression [95,96]. This protective effect is attributed to the stabilization of lipids in the cell membrane by polyamines, particularly spermine [97]. Thus, while AGM has beneficial effects on TJP colocalization, by preventing its own metabolism into antioxidant polyamines, increased availability of AGM may facilitate oxidative damage to surrounding cells, which is in line with current data, as well as literature cautioning against excessive AGM intake [47]. Current data clearly illustrate the importance of dose-regulation of AGM, as there seems to be a fine line between beneficial and detrimental effects.

In the context of AGM, an important role for rooibos has been demonstrated. Firstly, Dludla et al. (2020) recently reported that a specific rooibos formulation (a combination of aspalathin and PPAG—both present in notable amounts in the GRE utilised here) exerted anti-apoptotic characteristics specifically [98]. Secondly, the high polyphenol (i.e., also non-enzymatic antioxidant) content of GRE was likely mitigating the relative lack of polyamine-related antioxidant activity here. In terms of specific constituents of the GRE responsible for this benefit, one candidate would be quercetin, as the most ubiquitous polyphenolic flavonoid known to prevent against oxidative damage to DNA oligonucleotides by reactive oxygen species [99]—and which was concentrated in our GRE extract.

Taken together, current data expands on the literature implicating TYR as major trigger in gastrointestinal disorders by suggesting that manipulation of E2 (or its receptors) may provide therapeutic effect. Furthermore, our data suggest that combination treatment with AGM and GRE may have substantial benefit in the context of intestinal inflammation and barrier disruption. These benefits of GRE seem to be directly linked to it antioxidant and—to a lesser extent—its anti-inflammatory characteristics, rather than to a phytoestrogenic effect. These data warrant further treatment development in a robust in vivo model, so that limitations of cell culture models may be overcome.

## 4. Methods and Materials

### 4.1. Rooibos Preparation

An ethanol extract from unfermented (green) rooibos leaves (GRE) was kindly donated by Mr Roy van Brummelen (Van Brummelen Consultants, Pretoria, South Africa). A profile of the major constituents of the extract is presented in the supplementary material (Supplementary Figure S1). This extract was reconstituted in DMSO and diluted in cell culture media to final experimental concentration of 100 μg/mL.

### 4.2. Cell Culture Maintenance

Colon adenocarcinoma (HT-29) cells were kindly donated by Dr Tanya Davis. For general maintenance, cells were cultured in 5 mM D-galactose-supplemented, glucose-free RMPI (Gibco, Waltham, MA, USA, 11879-020) culture medium to facilitate differentiation. The culture medium was additionally supplemented with 10% heat-inactivated, gamma-irradiated fetal bovine serum (FBS) (Biowest, Nuaille, France, S181Y-500) and 1% PenStrep. Cells were sub-cultured with 1x trypsin and maintained in a humidified incubator at 5% CO_2_ at 37 °C. All experiments were done in triplicate and repeated at least three times. In addition, to optimize GRE dosage, cell viability utilising the WST-1 assay was performed (Supplementary Figure S2). Much higher (≈2- to 4-fold) doses of green rooibos extracts containing similar aspalathin content (≈20%) have been shown to reduce cell viability in HT-29 cells [56], which may have affected outcomes measured in this model. However, the dose employed in the current study was significantly lower, and no cell toxicity was evident (Supplementary Figure S2), so cytotoxicity is unlikely to be a confounder in data interpretation in this study.

### 4.3. Tight-Junction Protein Immunofluorescent Staining

In order to investigate the ability of GRE to mediate gut health, HT-29 cells were exposed to TA doses previously demonstrated to detrimentally affect TJP profile [37]. Briefly, HT-29 cells were seeded at 1.5 × 10^5^ cells/well (24-well plate) onto sterilized 12 mm round glass coverslips, which were previously coated with ECL cell-attachment matrix (Merck, Rahway, NJ, USA, 08-110) and refreshed every other day. After 8 days of culturing, cell monolayers were pre-treated with 100 μg/mL GRE, 1 nM E2, or a media vehicle (control). After 24 h of pre-treatment, the cell monolayers were treated with fresh medium containing 200 μg/mL TA (β-phenethylamine (PEA), tryptamine (TRP), ρ-tyramine (TYR) or agmatine (AGM)) or media vehicle (control) in the presence or absence of GRE (100 μg/mL) or E2 (1 nM) for an additional 24 h. The supernatants were collected for additional analyses, after which the cell monolayers were washed once with PBS. Monolayers were fixed (ice-cold 4% PFA and 50% MeOH solution) for 15 min at 20 °C, washed again with S-PBS (0.1% saponin in PBS), and blocked (20% FBS and 5% donkey serum in S-PBS) for 1 hr at room temperature. Overnight primary antibody incubations at 4 °C followed, utilising the primary antibodies: 1:250 mouse anti-ZO-1 (Invitrogen, Waltham, MA, USA, 33-9100) and 1:250 rabbit anti-occludin (Novus Biologicals, Littleton, CO, USA, NBP1-87402) in blocking buffer. Next, the monolayers were washed 3× with S-PBS, prior to incubation with secondary antibodies: 1:250 Alexa Fluor 488 donkey anti-mouse (Invitrogen, A-21202) and Alexa Fluor 594 donkey anti-rabbit (Invitrogen, A-21207) in blocking buffer for 1 hr at room temperature. Finally, the monolayers were incubated with Hoechst (ThermoFisher Scientific, Waltham, MA, USA, R37605) for 20 min before undergoing 4× washes with PBS. The glass coverslips were then mounted onto microscope slides with Dako Fluorescent Mounting Medium (Diagnostech, Johannesburg, South Africa, S3023). Fluorescently stained cell monolayers were imaged with the Carl Zeiss Confocal LSM 780 Elyra PS1 using the 60× oil-immersion objective. Respective Z-stack (10 slices) images were captured, and maximum intensity projections were analysed for co-localisation of ZO-1 and occludin utilising Zeiss ZEN imaging software.

### 4.4. Supernatant Analyses

Cell culture supernatant prostaglandin E2 (PGE2) concentrations were assayed using a commercially available enzyme-linked immunosorbent assay (ELISA) kit (E-EL-0034, Elabscience, Wohan, China), following the manufacturer’s guideline protocol. In addition, supernatants were analysed for IFN-γ, IL-1β, IL-6, IL-8, IL-10 and TNFα using a Human Magnetic Luminex Screening Assay (LXSAHM-06, R&D Systems, Minneapolis, MN, USA), following the manufacturer’s guideline protocol.

### 4.5. Statistical Analyses

All experiments were conducted in triplicate and repeated at least three times. Triplicate values were averaged to yield a final n = 3 for all data points presented. Statistical analyses of all data were completed utilising GraphPad Prism Version 9.1.2 (GraphPad Software LLC, San Diego, CA, USA). TJP data are represented qualitatively as representative images, as well as quantitatively as mean ± standard error of mean (SEM), while PGE2 and cytokine data are represented as means ± standard deviation (SD). Statistical analyses entailed 2-way ANOVAs with Tukey’s multiple comparison tests for TJP, PGE2 and cytokine data. A *p*-value of < 0.05 was considered statistically significant.

## Data Availability

Data is contained within the article and supplementary material.

## Supplementary Materials

The following supporting information can be downloaded at: https://www.mdpi.com/article/10.3390/ph15091097/s1, Figure S1: Changes in the relative distribution (% of total) of fifteen major phenolic constituents due to differential rooibos processing; Figure S2: WST-1 results showing the effect of varying green rooibos extract (GRE) doses on HT-29 colon cell viability [100,101,102,103,104,105,106,107,108,109,110,111,112,113,114,115,116,117,118,119,120].
