# Supplementary material for: Aspalathus linearis (Rooibos) and Agmatine May Act Synergistically to Beneficially Modulate Intestinal Tight Junction Integrity and Inflammatory Profile
Source: Pharmaceuticals (Basel). 2022 Sep 1;15(9):1097. doi: 10.3390/ph15091097 (PMC9501288; doi:10.3390/ph15091097)
Supplement: Supplementary file 1 [file pharmaceuticals-15-01097-s001.zip › pharmaceuticals-1860436-supplementary.pdf]

pharmaceuticals

MDPI

Supplementary material

Aspalathus linearis (rooibos) and agmatine may act synergistically to beneficially modulate intestinal tight junction integrity and inflammatory profile

L. Pretorius[1], & C. Smith[2*]

[1] Department of Physiological Sciences, Faculty of Science, Stellenbosch University, Stellenbosch, 7600, South Africa

[2] Division of Clinical Pharmacology, Department of Medicine, Faculty of Medicine and Health Sciences, Stellenbosch University, Cape Town, 7500, South Africa

* Corresponding author

Email: csmith@sun.ac.za

Postal address: Prof Carine Smith

Division Clinical Pharmacology

Dept Medicine

Faculty of Medicine and Health Sciences

Stellenbosch University

Private Bag X1

MATIELAND

7500

South Africa

Methods and materials

Quantification of phenolic constituents

We employed a previously described methodology [1] to quantify fifteen major phenolic constituents commonly found in rooibos tea. Briefly, tea samples were extracted with 50% MeOH and 1% formic acid (FA). A Waters Synapt G2 Quadrupole time-of-flight (q-TOF) mass spectrometer (MS) connected to a Waters Acquity ultra-performance liquid chromatograph (UPLC) was used for high-resolution UPLC-MS analysis (CAF, Stellenbosch University). Chromatographic separation was achieved on a Waters HSS T3 column (1.7 µm, 2.1 × 100 mm), and the column temperature was maintained at 55 °C. For all samples an injection volume of 2 µL was used and run using a binary mobile phase gradient which consisted of (A) 0.1% FA in H_2O and (B) 0.1% FA in Acetonitrile. The flow rate was set to 0.3 mL/min throughout the set 29 min run time with the following separation conditions: the gradient started at 0% solvent B for 1 min and increased to 28% B over 22 mins in a linear way. It was then increased to 40% solvent B until

22.5 min, followed by an increase to 100% solvent B until 23 min. Solvent B was held at 100% until 24.5 min, before being reduced to 0% solvent B to re-equilibrate to initial conditions for the final 4.5 min. Electrospray ionization was used in negative mode with a cone voltage of 15 V. Constituents were identified according to their accurate mass, MS/MS fragments, UV maxima and retention times as previously described [1] and quantified relative to rutin reference standards.

Choice of rooibos formulation

The decision to utilise a single rooibos preparation in this study (i.e. GRE) was manifold. Firstly, it is well-known that various factors influence the polyphenolic content, and subsequent antioxidant capacity, of rooibos. Some of these factors include geographical variety variations [1], season and quality grade [2], processing [3] and tea preparation i.e. brewing method [4]. As mentioned in the introduction, we have previously characterised 15 major polyphenolic constituents in green rooibos (GR), fermented rooibos and GRE (used in this study). Secondly, from these results (Supplementary Figure 1) we observed that the process of ethanol extraction concentrated constituents with well documented antioxidant activities. Indeed, orientin [5-7], iso-orientin [8-10], luteolin-7-O-glucoside [11-13], quercetin-3-O-robinobioside [14-16], and vicenin-2 [17-19] were all present in higher levels in GRE compared to the original GR stock, and have well-documented anti-oxidant activities. Thirdly, the specific antioxidant activity of GRE utilised here has previously been determined [20], which could be linked to outcome measures in this study. Fourthly, GRE also had higher phenylpropenoic acid glucoside (PPAG) levels than GR stock, and has been reported to confer protection downstream of oxidative stress induction [21], presumably via anti-apoptotic mechanisms [22]. Taken together, we suggest that there is scope for specific rooibos constituent compounding (selected bioactive compound combinations and distributions) to promote synergistic actions to obtain optimal therapeutic benefit. As such, we selected to utilise GRE in this study, as it represented the rooibos formulation with the most abundant distribution of a wide variety of polyphenolic constituents with known antioxidant and anti-apoptotic activities.

Choice of rooibos dose

Due to the substantial polyphenolic content [1,23] and documented antioxidant activity of GRE [20,24], it was important to establish a feasible dose to investigate in cell culture. Previous work executed on GRE by our group utilized a final experimental concentration of 100 µg/mL *in vitro*, which was determined to equate to an 8-fold concentration of rooibos

tea in terms of polyphenol content [20]. To determine the optimal dose of GRE for this current study, HT-29 cell viability was assessed in response to a range of GRE (0 to 1000 µg/mL). For this purpose, a WST-1 assay was used to assess the activity of mitochondrial enzymes (succinate-tetrazolium reductase system) in treated cells. Briefly, HT-29 cells were seeded in a 96-well microtiter plate with $4x10^4$ cells/well in phenol free media RMPI (Gibco, 11835-030) and were incubated at 37°C in 5% CO_2 after treatments. After a 24 hr incubation, 5 µL of WST-1 reagent (Abcam, ab155902) was added to each well and left to incubate for 90 min before the absorbance of each well was read on a plate reader (BIO-TEK, EL800) at 450 nm. Data were collected utilizing the KC Junior Software before exporting to Microsoft Excel for further analysis.

Results

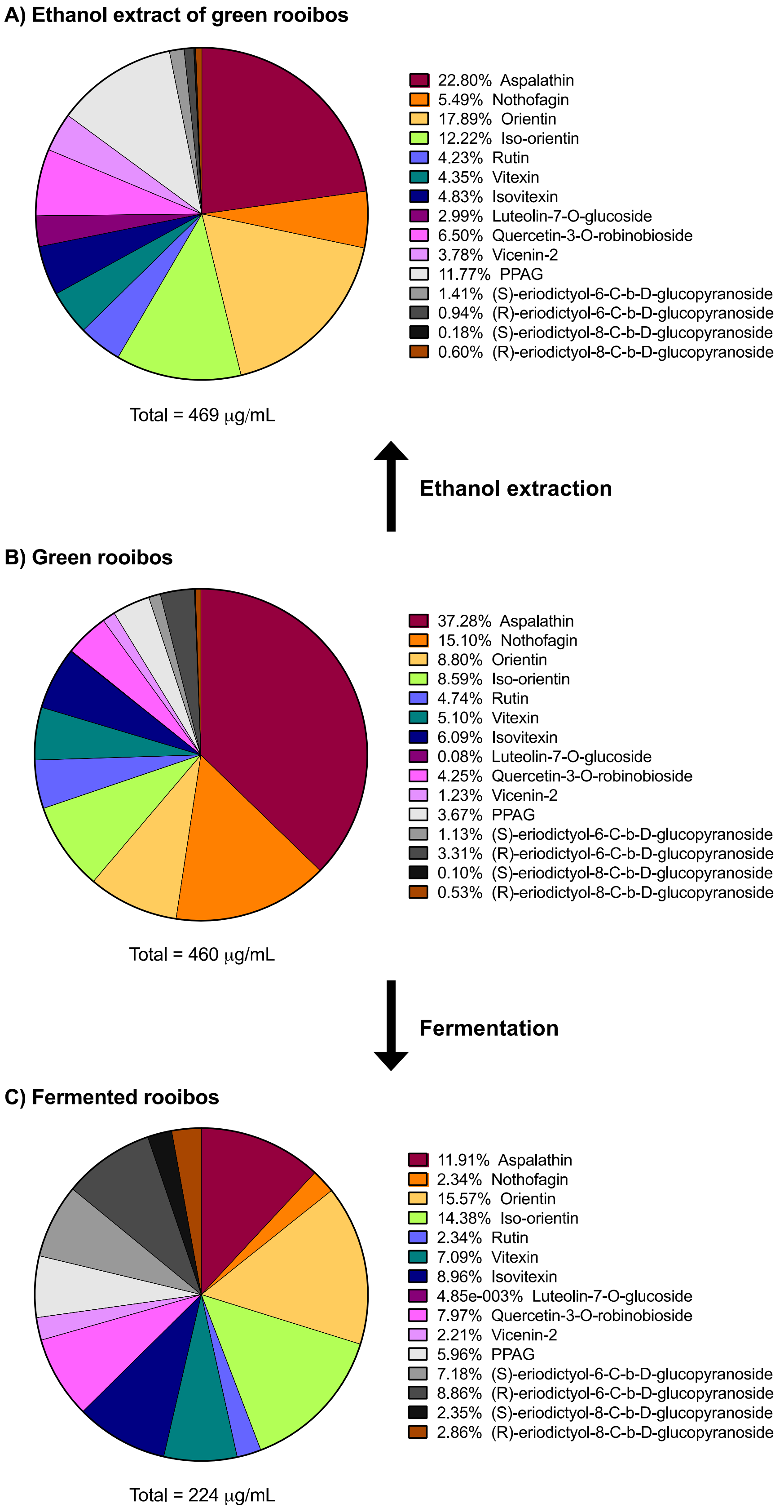
A) Ethanol extract of green rooibos
22.80% Aspalathin
5.49% Nothofagin
17.89% Orientin
12.22% Iso-orientin
4.23% Rutin
4.35% Vitexin
4.83% Isovitexin
2.99% Luteolin-7-O-glucoside
6.50% Quercetin-3-O-robinobioside
3.78% Vicenin-2
11.77% PPAG
1.41% (S)-eriodictyol-6-C-b-D-glucopyranoside
0.94% (R)-eriodictyol-6-C-b-D-glucopyranoside
0.18% (S)-eriodictyol-8-C-b-D-glucopyranoside
0.60% (R)-eriodictyol-8-C-b-D-glucopyranoside
Total = 469 μg/mL
Ethanol extraction
B) Green rooibos
37.28% Aspalathin
15.10% Nothofagin
8.80% Orientin
8.59% Iso-orientin
4.74% Rutin
5.10% Vitexin
6.09% Isovitexin
0.08% Luteolin-7-O-glucoside
4.25% Quercetin-3-O-robinobioside
1.23% Vicenin-2
3.67% PPAG
1.13% (S)-eriodictyol-6-C-b-D-glucopyranoside
3.31% (R)-eriodictyol-6-C-b-D-glucopyranoside
0.10% (S)-eriodictyol-8-C-b-D-glucopyranoside
0.53% (R)-eriodictyol-8-C-b-D-glucopyranoside
Total = 460 μg/mL
Fermentation
C) Fermented rooibos
11.91% Aspalathin
2.34% Nothofagin
15.57% Orientin
14.38% Iso-orientin
2.34% Rutin
7.09% Vitexin
8.96% Isovitexin
4.85e-003% Luteolin-7-O-glucoside
7.97% Quercetin-3-O-robinobioside
2.21% Vicenin-2
5.96% PPAG
7.18% (S)-eriodictyol-6-C-b-D-glucopyranoside
8.86% (R)-eriodictyol-6-C-b-D-glucopyranoside
2.35% (S)-eriodictyol-8-C-b-D-glucopyranoside
2.86% (R)-eriodictyol-8-C-b-D-glucopyranoside
Total = 224 μg/mL

Supplementary Figure S1: Changes in the relative distribution (% of total) of fifteen major phenolic constituents due to differential rooibos processing: (A) green rooibos ethanol extract (GRE), (B) green rooibos, and (C) fermented rooibos. Abbreviations: PPAG: phenylpropenoic acid glucoside.

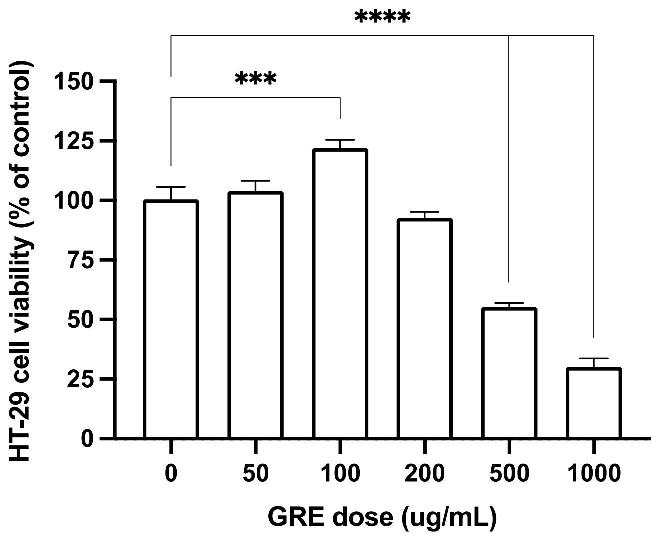

Supplementary Figure S2: WST-1 results showing the effect of varying green rooibos extract (GRE) doses on HT-29 colon cell viability. Data are represented as mean % of control ± SD, n=3. Statistical analysis: One-way ANOVA with Tukey's multiple comparison test, *** = $p<0.001$, **** = $p<0.0001$.

Results from the WST-1 assay suggested that 100 ug/mL of GRE had a beneficial effect on HT-29 cell viability ($p<0.001$), and as such was selected as the dose that was utilised in subsequent experimentation. Furthermore, these results suggest that an over-dose of GRE (>200 µg/mL), as with all antioxidants [25], can have detrimental effects on cell mitochondrial functionality and survival.

Additional references

1. Stander, M.A.; Van Wyk, B.E.; Taylor, M.J.C.; Long, H.S. Analysis of Phenolic Compounds in Rooibos Tea (Aspalathus linearis) with a Comparison of Flavonoid-Based Compounds in Natural Populations of Plants from Different Regions. *J Agric Food Chem* **2017**, *65*, 10270-10281, doi:10.1021/acs.jafc.7b03942.

2. Joubert, E.; Beelders, T.; de Beer, D.; Malherbe, C.J.; de Villiers, A.J.; Sigge, G.O. Variation in phenolic content and antioxidant activity of fermented rooibos herbal tea infusions: role of production season and quality grade. *J Agric Food Chem* **2012**, *60*, 9171-9179, doi:10.1021/jf302583r.

3. Pretorius, L.; Van Staden, A.D.; Kellermann, T.A.; Henning, N.; Smith, C. Rooibos (Aspalathus linearis) alters secretome trace amine profile of probiotic and commensal microbes in vitro. *J Ethnopharmacol* **2022**, *297*, 115548, doi:10.1016/j.jep.2022.115548.

4. Damiani, E.; Carloni, P.; Rocchetti, G.; Senizza, B.; Tiano, L.; Joubert, E.; de Beer, D.; Lucini, L. Impact of Cold versus Hot Brewing on the Phenolic Profile and Antioxidant Capacity of Rooibos (Aspalathus linearis) Herbal Tea. *Antioxidants (Basel)* **2019**, *8*, doi:10.3390/antiox8100499.

5. An, F.; Yang, G.; Tian, J.; Wang, S. Antioxidant effects of the orientin and vitexin in Trollius chinensis Bunge in D-galactose-aged mice. *Neural Regen Res* **2012**, *7*, 2565-2575, doi:10.3969/j.issn.1673-5374.2012.33.001.

6. Nayak, V.; Uma, P. Antioxidant and Radioprotective Effects of Ocimum Flavonoids Orientin and Vicenin in Escherichia coli. *Defence Science Journal* **2006**, *56*.

7. Praveena, R.; Sadasivam, K.; Deepha, V.; Sivakumar, R. Antioxidant potential of orientin: A combined experimental and DFT approach. *Journal of Molecular Structure* **2014**, *1061*, 114-123, doi:10.1016/j.molstruc.2014.01.002.

8. Cheel, J.; Theoduloz, C.; Rodriguez, J.; Schmeda-Hirschmann, G. Free Radical Scavengers and Antioxidants from Lemongrass (Cymbopogon citratus (DC.) Stapf.). *J. Agric. Food Chem.* **2005**, *53*, 2511-2517.

9. Sarıkahya, N.B.; Pekmez, M.; Arda, N.; Kayce, P.; Yavaşoğlu, N.Ü.K.; Kırmızıgül, S. Isolation and characterization of biologically active glycosides from endemic Cephalaria species in Anatolia. *Phytochemistry Letters* **2011**, *4*, 415-420, doi:10.1016/j.phytol.2011.05.006.

10. Yuan, L.; Li, X.; He, S.; Gao, C.; Wang, C.; Shao, Y. Effects of Natural Flavonoid Isoorientin on Growth Performance and Gut Microbiota of Mice. *J Agric Food Chem* **2018**, *66*, 9777-9784, doi:10.1021/acs.jafc.8b03568.

11. Park, C.M.; Song, Y.S. Luteolin and luteolin-7-O-glucoside protect against acute liver injury through regulation of inflammatory mediators and antioxidative enzymes in GalN/LPS-induced hepatitic ICR mice. *Nutr Res Pract* **2019**, *13*, 473-479, doi:10.4162/nrp.2019.13.6.473.

12. Rehfeldt, S.C.H.; Silva, J.; Alves, C.; Pinteus, S.; Pedrosa, R.; Laufer, S.; Goettert, M.I. Neuroprotective Effect of Luteolin-7-O-Glucoside against 6-OHDA-Induced Damage in Undifferentiated and RA-Differentiated SH-SY5Y Cells. *Int J Mol Sci* **2022**, *23*, doi:10.3390/ijms23062914.

13. Song, Y.S.; Park, C.M. Luteolin and luteolin-7-O-glucoside strengthen antioxidative potential through the modulation of Nrf2/MAPK mediated HO-1 signaling cascade in RAW 264.7 cells. *Food Chem Toxicol* **2014**, *65*, 70-75, doi:10.1016/j.fct.2013.12.017.

14. Novaes, P.; Torres, P.B.; Cornu, T.A.; Lopes, J.d.C.; Ferreira, M.J.P.; Santos, D.Y.A.C.d. Comparing antioxidant activities of flavonols from Annona coriacea by four approaches. *South African Journal of Botany* **2019**, *123*, 253-258, doi:10.1016/j.sajb.2019.03.011.

15. Xu, D.; Hu, M.J.; Wang, Y.Q.; Cui, Y.L. Antioxidant Activities of Quercetin and Its Complexes for Medicinal Application. *Molecules* **2019**, *24*, doi:10.3390/molecules24061123.

16. Zheng, Y.Z.; Deng, G.; Liang, Q.; Chen, D.F.; Guo, R.; Lai, R.C. Antioxidant Activity of Quercetin and Its Glucosides from Propolis: A Theoretical Study. *Sci Rep* **2017**, *7*, 7543, doi:10.1038/s41598-017-08024-8.

17. Duan, X.; Wu, T.; Liu, T.; Yang, H.; Ding, X.; Chen, Y.; Mu, Y. Vicenin-2 ameliorates oxidative damage and photoaging via modulation of MAPKs and MMPs signaling in UVB radiation exposed human skin cells. *J Photochem Photobiol B* **2019**, *190*, 76-85, doi:10.1016/j.jphotobiol.2018.11.018.

18. Lee, B.-S.; Yang, S.; Lee, C.; Ku, S.-K.; Bae, J.-S. Renal protective effects of vicenin-2 and scolymoside in a mouse model of sepsis. *Brazilian Journal of Pharmaceutical Sciences* **2020**, *56*, doi:10.1590/s2175-97902019000418636.

19. Li, Y.; Zheng, Y.; Wang, H. Anticancer activity of Vicenin-2 against 7,12 dimethylbenz[a]anthracene-induced buccal pouch carcinoma in hamsters. *J Biochem Mol Toxicol* **2021**, *35*, e22673, doi:10.1002/jbt.22673.

20. Lopez, V.; Casedas, G.; Petersen-Ross, K.; Powrie, Y.; Smith, C. Neuroprotective and anxiolytic potential of green rooibos (Aspalathus linearis) polyphenolic extract. *Food Funct* **2022**, *13*, 91-101, doi:10.1039/d1fo03178c.

21. Himpe, E.; Cunha, D.A.; Song, I.; Bugliani, M.; Marchetti, P.; Cnop, M.; Bouwens, L. Phenylpropenoic Acid Glucoside from Rooibos Protects Pancreatic Beta Cells against Cell Death Induced by Acute Injury. *PLoS One* **2016**, *11*, e0157604, doi:10.1371/journal.pone.0157604.

22. Dludla, P.V.; Muller, C.J.F.; Louw, J.; Mazibuko-Mbeje, S.E.; Tiano, L.; Silvestri, S.; Orlando, P.; Marcheggiani, F.; Cirilli, I.; Chellan, N.; et al. The Combination Effect of Aspalathin and Phenylpyruvic Acid-2-O-beta-D-glucoside from Rooibos against Hyperglycemia-Induced Cardiac Damage: An In Vitro Study. *Nutrients* **2020**, *12*, doi:10.3390/nu12041151.

23. Iswaldi, I.; Arraez-Roman, D.; Rodriguez-Medina, I.; Beltran-Debon, R.; Joven, J.; Segura-Carretero, A.; Fernandez-Gutierrez, A. Identification of phenolic compounds in aqueous and ethanolic rooibos extracts (Aspalathus linearis) by HPLC-ESI-MS (TOF/IT). *Anal Bioanal Chem* **2011**, *400*, 3643-3654, doi:10.1007/s00216-011-4998-z.

24. Marnewick, J.L. Antioxidant Properties of Rooibos (Aspalathus linearis) – In Vitro and in Vivo Evidence. In *Systems Biology of Free Radicals and Antioxidants*; 2014; pp. 4083-4108.

25. Joubert, E.; Winterton, P.; Britz, T.J.; Gelderblom, W.C.A. Antioxidant and Pro-oxidant Activities of Aqueous Extracts and Crude Polyphenolic Fractions of Rooibos (Aspalathus linearis). *J. Agric. Food Chem.* **2005**, *53*, 10260−10267.
